# Translating Research as an Approach to Enhance Science Engagement

**DOI:** 10.3390/ijerph15081749

**Published:** 2018-08-15

**Authors:** Michelle T. Juarez, Chloe M. Kenet

**Affiliations:** City University of New York School of Medicine, City College of New York, New York, NY 10031, USA; chloestingray@gmail.com

**Keywords:** science communication, translation, education, frontiers for young minds, health literacy

## Abstract

The impact of research depends on the effective communication of discoveries. Scientific writing is the primary tool for the dissemination of research, and is an important skill that biomedical trainees have to develop. Despite its importance, scientific writing is not part of the mainstream curriculum. One strategy used to teach scientific writing is holding a journal club style discussion of primary research literature that the students are asked to read. However, this activity can result in a passive learning experience and limit the development of trainees’ scientific writing skills. In order to improve trainees’ written communication skills, we tested an exercise that involved generating a revised article describing prior research, in essence “translating” the science into basic language. Following the guidelines set out by “Frontiers for Young Minds” and feedback received from “Young Reviewers”, we wrote a revised article with a simpler description of the research. In this article, we describe this scientific writing exercise, which may ultimately serve as a model for scientists to share their research more efficiently in order to promote better public health outcomes.

## 1. Introduction

Health literacy is a fundamental concept in public health that links policy issues in education with outcomes in the health care system [1]. A defined set of health education standards establishes a framework for health literacy concepts to align them with student learning benchmarks [2]. Recent studies have correlated health status with literacy skills, with a particular emphasis on the comprehension of health-related information [3]. Since scientific articles are a source of such information, an interesting hypothesis can be put forth—if biomedical articles are translated into a language that the public can easily understand, then health interventions may be more effective. The authors of these publications, namely scientists and health professionals, would be best suited as translators, but the challenge is to refine the language into a simpler description of the science. The goal of our research was to highlight a pilot project that applied a revised scientific article as a tool to promote science communication skill development in biomedical research trainees. We recruited students to help filter the scientific message. We discovered that the learning issues faced by trainees reading a new concept in a scientific article provided insight into how to describe the fundamental knowledge discovered in the research. To complement the communication skill development, we also focused on a few strategies that enhanced science learning. Using techniques such as the teaching of science as a second language has demonstrated positive gains in science learning [4]. The inclusion of artistic techniques (e.g., drawing a representation of the scientific concepts) can blend multiple modes of learning, namely reasoning, communication, and engagement, to improve scientific literacy [5]. A synergistic approach of integrating inquiry into the students’ exposure to scientific concepts is a productive method of developing literacy in science [6]. Exploring these science learning strategies with our trainees led to an additional hypothesis—science learning techniques may reflect a method of improving community health literacy. In this article, we propose that health advances can have an impact on the community if more scientists generate revised versions of their research articles with simpler language, using a Frontiers for Young Minds style, for example.

Research trainees often try to supplement their science learning with new concepts from scientific articles [7,8]. However, this strategy can be challenging for trainees because of the specialized style of writing often used in these articles. When new students joined our team for an independent studies course or a summer research internship, their first task was to read a few scientific articles. We then used a journal club style discussion to ask the students questions about their understanding of the concepts. In the past, when the students read the assigned articles, they often were not able to assess the experiments’ significance because of a lack of context for the studies. Reading scientific articles requires more critical thinking than reading other types of articles. The reader needs to be able to understand the data and compare the results with previous studies. In the majority of cases, reading scientific articles requires prior knowledge because they are usually written for a specialized, well-informed audience, who is inherently expected to possess information about the topics.

It often takes a considerable amount of time to read a scientific article. Furthermore, due to the restrictions on article length imposed by scientific journals, authors are often forced to keep explanations brief. As trainees began to read a new article, their struggle to understand the subject was compounded by the content density. In addition to looking up new concepts and technical language, much time was spent trying to understand the general writing style. Though it was possible to get through an article quickly, much more time was needed to truly read it in depth. Research trainees developed a routine to navigate and absorb the contents of the article more efficiently. For example, they started with the published date, the title of the article, and the abstract/introduction to get an overview of what to expect in the article. Then, they read the discussion and conclusion sections to understand what the results of the article were. In order to gain an understanding of the study logistics, they then read the materials and methods and the results sections, which provided additional details. Overall, reading a science article was a learning experience.

Research trainees need to practice reading science articles, as they do with other aspects of science. After some practice, a pattern of organization begins to emerge in every article. It becomes easier to interpret the information after recognizing this pattern. With more practice, it is possible to comment internally about the meaning of the data provided or whether they align with the study’s hypothesis. Once the key information is understood, it is often easier to read the article and then apply the information.

## 2. Materials and Methods

The material used for this project was an article published on our research [9]. The article summarized a genetic screen used to identify new factors that controlled a reaction to injury in the fruit fly. A genetic screen is a method of searching through a large collection of genetically different samples to find genes responsible for a reaction to a test (e.g., looking for a needle in a haystack). The results of our genetic screen were detected using a color reporter—a tool used to visualize the reaction to injury under a microscope.

The method for this project was a translation of the genetic screen article, with the goal of promoting research trainee communication skills. An additional resource for our translation project was a methods article that was published as a video [10]. For our translation project, we followed the guidelines of Frontiers for Young Minds—an online journal that publishes reimagined versions of previously published and peer-reviewed articles (https://kids.frontiersin.org/). This translation project required minimal resources. There was no cost associated with publishing the article and our timeline covered approximately 15 h of group discussions spread out over one semester.

The Frontiers for Young Minds journal serves as an open-access resource that not only creates scientific literature for a broad audience, but also brings kids into the review process. Specifically, scientists write a basic version of their article, which is then reviewed by young people in the target age range (ages 8–15). Authors “translate” the main ideas in the article through the use of keywords as well as a glossary section to define relevant scientific nomenclature. A science mentor—other than the authors—guides the young reviewers through the review process. In an online discussion forum hosted by the Frontiers for Young Minds editors, the authors and mentors discuss the comments from the young reviewers, and work together to identify components of the articles that sparked the kids’ curiosity and concepts that needed further clarification.

**Figure 1:**
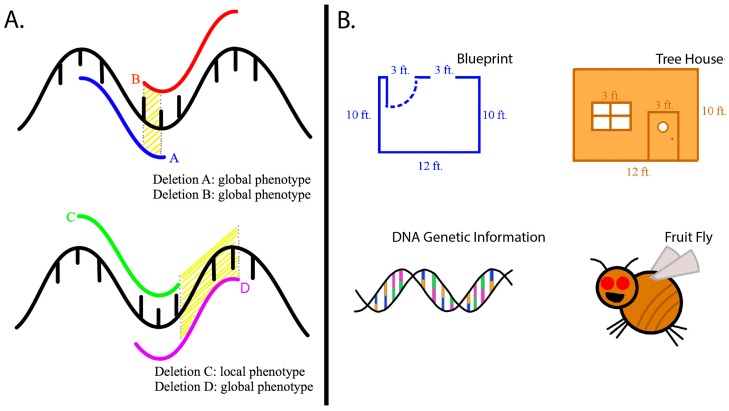
Main focus of the Frontiers for Young Minds article. (**A**) Initial illustration. (**B**) Revised illustration.

**Figure 2:**
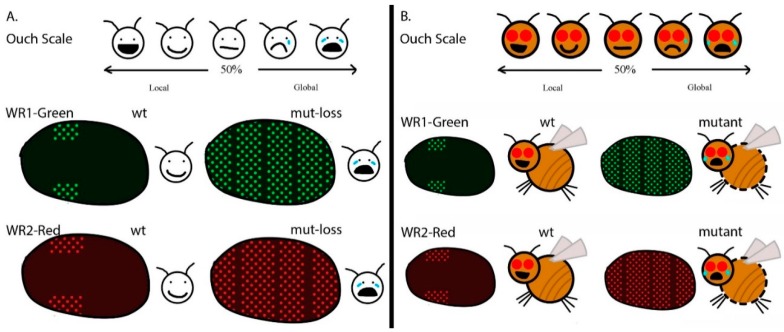
Highlights of the fruit fly in creative illustrations. (**A**) Simple “ouch scale” illustration. (**B**) Enhanced “ouch scale” illustration.

For our translation project, we chose three figures to represent the main focus of the study. The results we wanted to illustrate were the genetic screen, the wound reporter pattern, and the mutant or chemical analyses [9]. In the first version of our revised article, we drew DNA and deletion images to highlight the concept of the genetic screen (see Figure 1A). We created an “ouch scale” to represent the wound reporter pattern. We drew embryos with corresponding “ouch scale” levels to represent the reactions of the wound reporter (see Figure 2A).

The young reviewers did not understand the deletion figure. They wanted to see a fruit fly and had questions about mutations. In the revised text, we highlighted a reference to a video that demonstrated the methods [10]. We redrew the figures to make an analogy with DNA and genetic information (see Figure 1B, Ref. [11]). We made an additional figure to explain the changes in the DNA and phenotypes in the fruit fly. In all three figures we included a fruit fly image to link the concepts together (see Figure 2B, Ref. [11]). The new translated article served as a resource to share with trainees that joined a research group and the community interested in learning more about science and health [11] (https://kids.frontiersin.org/article/10.3389/frym.2016.00027).

## 3. Results

Over the past two years, after working with 15 trainees on translating an article and then reading the companion research article, a small cohort was generated to help us observe results. From the trainees’ comments about the writing and reading experience, reflections emerged on the impact this assignment had on their scientific literacy. It was challenging to determine the bigger picture of the research article, and trainees became distracted by concentrating on a specific aspect of the research. Although Frontiers for Young Minds was intended for younger audiences, trainees who read this style of article had a better understanding of the research goals. Frontiers for Young Minds also presented a good model for how research trainees could approach reading scientific articles and create a narrative out of the information presented in the article. Learning to focus on the scientific questions provided the trainees with a platform to investigate why a certain experiment was performed and how it made a scientific argument more persuasive.

As previously mentioned, reading scientific articles requires some scientific knowledge; therefore, using the “ouch scale” for comparing scientific data was very useful, especially when the audiences were kids (see Figure 2B). The new article provided a great summary for younger students. Terms were defined and concepts were explained, so it was not left to the reader to decipher or look up. The collaboration between the authors, science mentor, and young reviewers aimed to make the science clear and easily understandable. Using images to portray complex ideas is much better than using text because not everyone is fluent in English or understands text in the same way. In addition, images in scientific articles encourage people to read them more, compared to text-only articles.

Even if an individual is reading about a new and complex research topic, it will be easier to follow the rest of the story once they understand the key information. This is exactly the goal of the Frontiers for Young Minds journal. It provided a foundation for the concepts in the simplest of terms. Research trainees benefited from switching between reading the translated version and the actual research article to solidify the information. The Frontiers for Young Minds became a kind of refresher article to be read before tackling the original science article. The trainees could make sure they were on the right path. They gained exposure to new fields with a quick reading assignment that was valuable for both reviewing and finding more information.

## 4. Discussion

A Frontiers for Young Minds-style writing project can be applied to learning environments beyond the research lab. By cultivating the creativity of our trainees, we explored the language of science within a new context and developed a stronger message to share with the public. Ultimately, the goal of this project was to improve science communication for a general audience. The focus of this short communication article was to describe an experience translating a science article. The lessons learned highlighted how a reader looked at a figure and how the choice of words helped to convey the scientific idea. The trainees reading the translated article thought more critically about experimental organization and became more critical about reading other scientific articles. 

**Figure 3:**
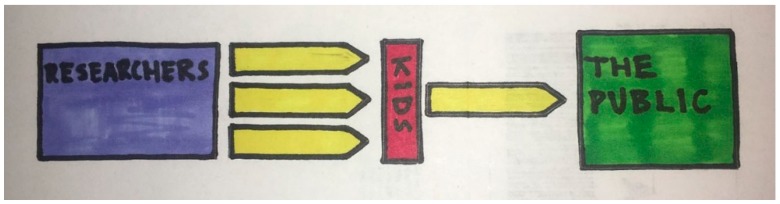
Information flow from Researchers to The Public. Kids—reviewers act as a filter to focus the information that researchers want to share with the public.

In addition to the science communication outcome of this translation project, we fostered a new connection between scientists and young minds. This stronger partnership will enable society to become an active participant in future science discoveries. One of the many benefits of carrying out a translation project with a young person is that they readily ask questions because of their natural curiosity, unbiased opinions, and enthusiasm. Through the eyes of the young reviewer, it is possible to refine the content of a research article and appeal to a broader audience (see Figure 3). This component of the Frontiers for Young Minds review process enables the basic discovery of a research article to be crafted into a message that may have a better impact on the public. In particular, the multi-generational collaboration forges an important bridge between two groups that are not often provided with opportunities to interact, and can broaden the scope of participatory action research [12]. This exercise in science communication can be a feasible method for public health professionals to expand the impact of their research and promote health literacy. The challenge of translating a research article will provide a powerful platform to share additional resources with the community. In addition, an article that has been translated into basic format can provide a better version for conversion into other languages.

Building on the capacity of our institutions and on the knowledge of our professional community, there are many ways to develop a Frontiers for Young Minds writing exercise. In this article, the trainees’ comments initiated the science translation process. In other non-academic settings, with limited access to trainees, an alternative method may be to engage the administration and support staff as collaborators to translate the research article. The writing collaborators serve as ambassadors to share the knowledge with their families and community. In both strategies, a new audience gains access to scientific discoveries. Translating research discoveries may also provide funding agencies that support research projects with additional products for dissemination. Here is an example of writing for the public: those who wish to obtain grant support from the National Institutes of Health are required to submit a brief project narrative. Therefore, one suggestion is that granting agencies require a summary of the research outcomes written in the Frontiers for Young Minds style, shared with the public.

With the increased translation of scientific innovations, written by the authors that produced the original research, we could foster more engagement between the scientists and the community. A potential outcome of this community engagement could be an improvement in the public support for research. Reflecting upon this science translation project, a case can be proposed that such a writing exercise may serve as a feasible model to expand the impact of scientific discoveries and share knowledge with society. Improving science communication will not only benefit scientific training, but also cultivate scientific literacy within communities having limited access to science and health fields.
